# Pseudophakic ametropia management with toric implantable collamer lens with a central hole (case report)

**DOI:** 10.1186/s12886-017-0414-6

**Published:** 2017-02-21

**Authors:** Xun Chen, Xiaoying Wang, Xingtao Zhou

**Affiliations:** 1 Myopia Key Laboratory of the Health Ministry, Shanghai, China; 2 EYE & ENT Hospital of Fudan University, Shanghai, China

**Keywords:** Implantable collamer lens, TICL V4c, Pseudophakic ametropia, Case report

## Abstract

**Background:**

To report the clinical outcomes of correcting pseudophakic ametropia using toric implantable collamer lens with a 360 um central hole (TICL V4c).

**Case presentation:**

The right eye of a 22-year-old male patient developed high myopia after unilateral phacoemulsification and intraocular lens (IOL) implantation following traumatic cataract 16 years ago. The manifest refraction was -11.50 DS/-2.50 DC × 175 with an uncorrected distance visual acuity (UDVA) of 20/2000 and a corrected distance visual acuity (CDVA) of 20/20. The manifest refraction of left eye was -6.25 DS/-3.75 DC × 180 with UDVA 20/200 and CDVA 20/20. Both eyes were implanted posterior chamber TICL V4c lens. Postoperatively, the refractive errors were +1.00 DS/-0.50 DC × 50 with UDVA 20/16 and CDVA 20/16 in the right eye and +0.75 DS/-0.75 DC × 45 with UDVA 20/16 and CDVA 20/13 in the left eye, respectively. No complications were observed.

**Conclusions:**

TICL V4c is safe, effective and predictable in managing pseudophakic ametropia.

## Background

Pseudophakic ametropia can be corrected by spectacles, contact lenses, intraocular lens (IOL) supplementation or exchange [1, 2], and corneal refractive surgeries [3, 4]. Spectacles offer an inconvenient approach and contact lenses have potential risks of dry eye and infectious keratitis. IOL exchange is a difficult option with potential complications of capsule tear, vitreous loss, and retinal detachment, especially if the lens is adherent to the capsular bag [2]. Corneal refractive surgeries can be used to correct lower amount of refractive errors when corneal thickness is in the safe range, but corneal scarring or haze, flap complications and regression may be the complications [3, 4]. In cases of higher refractive errors, the new posterior chamber implantable collamer lens (ICL V4c) provides an alternative approach, as corneal thickness may not meet the requirements of large refractive corrections. The new 360 μm central hole design of ICL V4c allows for the natural flow of aqueous humor without the need for a peripheral iridotomy [5]. This study investigates the clinical outcomes of correcting high myopia of a young adult in the management of pseudophakic ametropia using TICL V4c.

## Case presentation

**Fig. 1:**
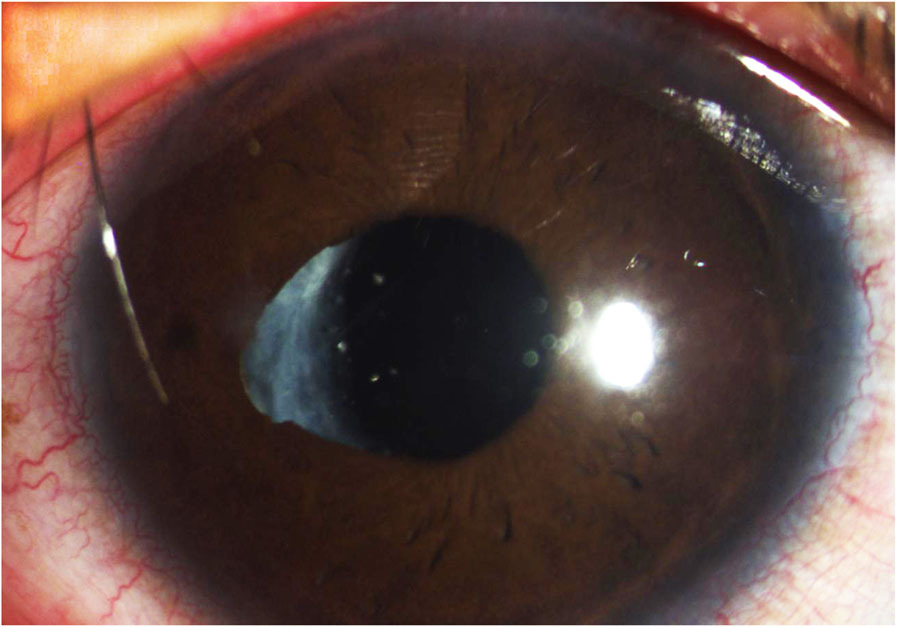
The preoperative anterior segment picture of the right eye

**Fig. 2:**
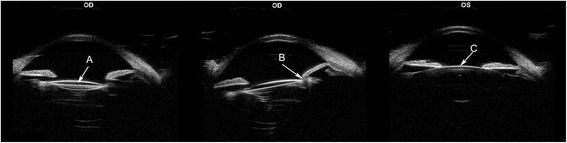
Preoperatively, ultrasound biomicroscopy (UBM) shows the IOL in the capsular bag of the right eye (**a**), adhesion between the temporal and subtemporal pupillary margin and the anterior capsule (**b**), and the crystal lens of the left eye (**c**)

The right eye of a 22-year-old male patient developed high myopia after unilateral phacoemulsification and intraocular lens (IOL) implantation following traumatic cataract 16 years ago and the patient had an urgent desire to get rid of the spectacles. A comprehensive ophthalmic examination was performed preoperatively in Eye and ENT Hospital of Fudan University. An IOL in the capsular bag with posterior capsular opacification was observed in the right eye, as well as corneal scar and oval pupil with pupillary margin adhesion (Fig. 1). No other disease was found in the remaining examination. Ultrasound biomicroscopy (UBM, 50 MHZ, AVISO V:4.0.2, Quantel Medical, France) shows the IOL in the capsular bag of the right eye, adhesion between the temporal and subtemporal pupillary margin and the anterior capsule, the crystal lens of the left eye and all suspensory ligament of both eyes in position (Fig. 2).

Preoperative data was summarized in Table 1. The manifest refraction was -11.50 DS/-2.50 DC* 175 in the right eye and -6.25 DS/-3.75 DC* 180 in left, with the CDVA being 20/20 in both eyes. The patient was not a suitable candidate for corneal laser refractive surgeries because of the relatively thin cornea (479, 478 microns, respectively). Then ICL was implanted in the in the sulcus of both eyes, leaving the previously implanted IOL in the capsular bag of the right eye. ICL power calculations were performed by the manufacturer (STAAR Surgical) using a modified vertex formula. The size (length) of the implanted ICL was determined based on the patient’s anterior chamber depth (ACD) and white-to-white (WTW), sulcus to sulcus (STS). The ICL power of the left eye was -10.5/+3.5/090, with the length being 12.6 mm and the residual spherical equivalent (SE) being -0.05 D. The selection of the ICL size was a key and difficult point for the pseudophakic eye due to the fact that the ACD enlarged after cataract surgery and IOL implantation. Therefore, the ACD of the left eye was a reference and the ICL size of right eye referred to that of the left eye. Then the power of the left eye was -10.5/+3.5/090, with the length being 12.6 mm and the residual SE being 0.00 D. The good position of ICL with ideal vault in the phakic eye suggests an accurate ICL size, then the ICL was implanted in the pseudophakic eye 1 week after the surgery of phakic eye.

**Table 1:** Preoperative Data

Eye	Manifest refraction	UDVA	CDVA	WTW(mm)	VSTS(mm)	HSTS(mm)	ACD (mm)	Keratometry (D)	ECD (cells/mm^2^)	CCT (um)
R	-11.50/-2.50 × 175	0.01	1.0	11.5	11.50	11.33	4.66	43.1/45.8 × 172.8	3341	479
L	-6.25/-3.75 × 180	0.1	1.0	11.6	11.48	11.38	3.08	43.2/46.8 × 172.0	3992	478

*R* right, *L* left, *UDVA* uncorrected distance visual acuity, *CDVA* corrected distance visual acuity, *VSTS* Vertical sulcus to sulcus, *HSTS* Horizontal sulcus to sulcus, *ACD* anterior chamber depth, *ECD* endothelial cell density, *CCT* central corneal thickness

### Surgical Procedure

The medications used 3 days before surgery were the same as the standard implantation of the lens [6, 7]. After cycloplegic agents (1% Tropicamide, Alcon, Belgium) and topical anaesthesia (0.4% Oxybuprocaine hydrochloride, Santen, Japan), the visco surgical device (Provisc, Alcon, Belgium) was placed into the anterior chamber and synechiolysis of pupil was performed with surgical scissors carefully, then the implantation of ICL and the remaining procedure were the same as our previous studies [6, 7].

### Follow-up

**Fig. 3:**
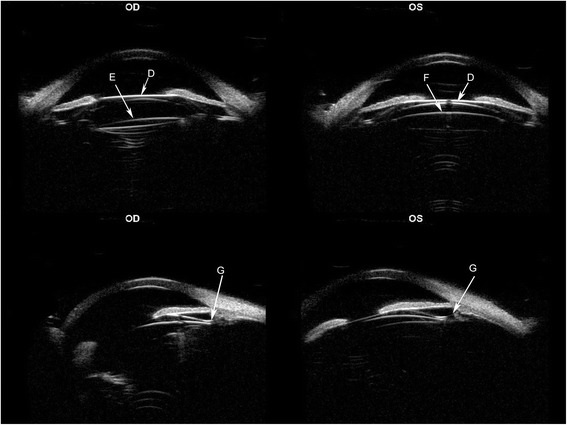
Postoperatively, ultrasound biomicroscopy(UBM) shows the ICL V4c implanted in both eyes(D), the primary IOL of the right eye(E), the crystal lens of the left eye(F). All the ICL haptics of both eyes were in ciliary crown(G)

The surgeries were uneventful and no intraoperative complication was observed. Postoperatively, slitlamp examination of both eyes showed a quiet anterior chamber and that the ICL was in the sulcus with the IOL in the capsular bag. The manifest refraction of the right eye was +1.00 DS/-0.50 DC × 45 with UDVA 20/16 and CDVA 20/16. The manifest refraction of the left eye was +0.50 DS/-0.75 DC × 30 with UDVA 20/16 and CDVA 20/16. UBM showed the ICL V4c implanted in both eyes, the 4 ICL haptics of right eye were placed in supernasal, nasal, temporal and subtemporal ciliary crown, respectively. The 4 ICL haptics of left eye were placed in supertemporal, temporal, nasal and subnasal ciliary crown, respectively. All angles of both eyes were open (Fig. 3).

Three months after TICL V4c implantation, the refractive errors were +1.00 DS/-0.50 DC × 50 with UDVA 20/16 and CDVA 20/16 in the right eye, +0.75 DS/-0.75 DC × 45 with UDVA 20/16 and CDVA 20/13 in the left eye, respectively. The intraocular pressure, the vault and the endothelial cell density of the right and the left eye were 15.3 mmHg(R), 14.8 mmHg (L), 880 um(R), 530 um (L), 3125 cells/mm^2^(R), 3940 cells/mm^2^ (L), respectively (Table 2).

**Table 2:** Postoperative Data

	Eye	Manifest refraction	UDVA	CDVA	IOP (mmHg)	Vault (um)	ACD (mm)	Keratometry (D)	CCT (um)	ECD (cells/mm^2^)
1 day	R	_	1.0		13.9	790	2.26	43.3/45.2 × 165.3	503	_
	L	_	1.0		18.4	500	2.26	41.1/46.8 × 179.1	481	_
1 week	R	+1.00/-0.50 × 45	1.2	1.2	16.6	820	2.33	43.5/45.5 × 165.6	490	_
	L	+0.50/-0.75 × 30	1.2	1.2	16.5	510	2.18	42.1/46.0 × 174.0	483	_
1 month	R	+1.25/-0.75 × 45	1.0	1.2	14.7	910	2.72	43.3/45.8 × 173.6	496	_
	L	+0.50/-0.75 × 30	1.2	1.2	15.6	660	2.29	42.1/46.2 × 173.7	490	_
3 month	R	+1.00/-0.5 × 50	1.2	1.2	15.3	880	2.69	43.3/45.4 × 170.1	495	3125
	L	+0.75/-0.75 × 45	1.2	1.5	14.8	530	2.30	42.2/45.9 × 171.3	491	3940

*R* right, *L* left, *UDVA* uncorrected distance visual acuity, *CDVA* corrected distance visual acuity, *IOP* intraocular pressure, *ACD* anterior chamber depth, *ECD* endothelial cell density, *CCT* central corneal thickness

## Discussion

Patients’ desire and their corneal conditions determine the options for correction of a refractive error. This patient had an urgent desire to get rid of the spectacles. Corneal refractive surgery is not an appropriate surgical correction for this patient because of his thin cornea. Lens replacement, on the other hand, is a difficult and risky surgical option because it has been a long time since the primary operation and that oval pupil with pupillary margin adhesion has been formed. Under the circumstances, ICL implantation has become the most appropriate choice.

Intraocular lens exchange for the correction of pseudophakic ametropia is feasible if the surgery is performed early. It would be difficult to replace an IOL into the bag, if anterior and posterior lens capsules were adhered to each other after a long-term primary surgery. Once the capsule shrinks around the IOL, complications such as capsule tear, vitreous loss, and retinal detachment may occur [2].

Corneal refractive surgery such as laser-assisted subepithelial keratomileusis (LASEK), laser-assisted in situ keratomileusis (LASIK) and small incision lenticule extraction (SMILE) is an option to correct pseudophakic ametropia [3, 4]. The procedures are irreversible and the incidence of complications such as flap complications and regression is well known. Lots of studies [8–10] report the ICL as a more favorable option than corneal refractive surgery in terms of higher stability and visual quality and its superior performance on dry eyes.

Implantation of supplementary lens for the correction of residual refractive error in pseudophakic eye is another option. Anterior chamber IOLs may cause endothelial cell loss and need a larger incision for insertion, as well as problems with pupil ovaling [11]. The technique of implanting 2 IOLs in the posterior chamber was described as “piggyback”, the traditional piggyback referred to that a conventional in-the-bag IOL was implanted in pseudophakic eye, which may cause interlenticular opacities because two IOL optics are placed close to each other [1, 12, 13]. Postoperatively, there were risks of intraocular pressure increase, IOL shift and pupil capture [12–14]. ICL compare favorably with IOL as the enough vault between the ICL and the primary IOL may contribute to decreasing the opacity [15–18]. Also, Toric ICL can correct the astigmatism. However, inappropriate ICL may cause TICL rotation or positioning error in pseudophakic eyes which can bring new astigmatism. Patients with severe vision loss or ghost after TICL rotation need adjustment of TICL position. The previous ICL V4 has a risk of postoperative intraocular pressure increase. At present, the ICL V4c decreases the rate of opacity and intraocular pressure increase without peripheral iridotomy for the central hole design [19]. Besides, pupil capture rarely occurs as the ICL haptics were placed in the ciliary sulcus. The accuracy of ICL is higher, because the calculation of ICL mainly depends on refraction, while the calculation of IOL mainly depends on the biometry parameters.

The option of ICL size is a problem for pseudophakic eyes. Both the ICL length evaluation and the power calculation were originally designed for phakic eyes, and it hasn’t been affirmed whether ICL would introduce significant error in pseudophakic eyes. Fortunately, the other phakic eye has not been implanted IOL and crystalline lens were reserved, and the parameters of the pseudophakic eye were similar with phakic eye except ACD. Therefore, the selection of ICL size in phakic eye was a reference to pseudophakic eye. Postoperatively, the ideal vault of both eyes was obtained. In the Takashi Kojima’s report [16], size of the ICL was chosen based on WTW and ACD, 6 eyes (75%) showed high vault (>3/2CT) after surgery, but scheimpflug image evaluation showed that the angle was open and the postoperative intraocular pressure was normal in these eyes with high vault. We believe that accurate ciliary sulcus diameter measured by UBM is necessary for the selection of ICL size. In general, the ACD of pseudophakic eye is far deeper than the phakic eye because the anterior surface of an IOL is situated further back in a pseudophakic situation than the that of the crystalline lens in a phakic situation. We advise that the ACD of phakic eye population may be a reference when choosing the size of ICL in pseudophakic eye if there was not a referable contralateral phakic eye.

## Conclusions

Our case suggests that TICL V4c is safe, effective and predictable in managing pseudophakic ametropia.

## Abbreviations

ACD: Anterior chamber depth
CCT: Central corneal thickness
CDVA: Corrected distance visual acuity
DC: Diopters cylinder
DS: Diopters sphericity
ECD: Endothelial cell density
HSTS: Horizontal sulcus to sulcus
ICL: Implantable collamer lens
IOL: Intraocular lens
IOP: Intraocular pressure
L: Left
LASEK: Laser-assisted subepithelial keratomileusis
LASIK: Laser-assisted in situ keratomileusis
R: Right
SE: Spherical equivalent
SMILE: Small incision lenticule extraction
STS: Sulcus to sulcus
TICL: Toric implantable collamer lens
UBM: Ultrasound biomicroscopy
UDVA: Uncorrected distance visual acuity
VSTS: Vertical sulcus to sulcus
WTW: White-to-white
